# The Relationship Between Periodontal Disease (PD) and Recurrent Vascular Events in Ischemic Stroke/Transient Ischemic Attack (TIA) Patients: A Hospital-Based Cohort Study

**DOI:** 10.7759/cureus.36530

**Published:** 2023-03-22

**Authors:** Anupriya Sharma, Ashish Sharma, Rakesh Chauhan, Abhimanyu S Chauhan, Monika Parmar, Sucheta Thakur

**Affiliations:** 1 Dentistry, Dr. Radhakrishnan Government Medical College, Hamirpur, IND; 2 Neurology, All India Institute of Medical Sciences, Bilaspur, IND; 3 Medicine, Dr. Radhakrishnan Government Medical College, Hamirpur, IND; 4 Oral and Maxillofacial Surgery, Himachal Pradesh Government Dental College & Hospital, Shimla, IND

**Keywords:** high periodontal disease, low periodontal disease, recurrent vascular events, periodontal disease, stroke/tia

## Abstract

Background

In this cohort study, the association between periodontal disease (PD) and recurrent vascular events was determined among the subjects with ischemic stroke/transient ischemic attack (TIA), and the extent and severity of periodontal disease were estimated among these subjects.

Methods

This prospective, longitudinal, hospital-based cohort study included 153 individuals who had a stroke or TIA. They were divided into two groups: high periodontal disease (HPD) (N=55, mean age: 59.40±12.21) and low periodontal disease (LPD) (N=98, mean age: 53.03±12.82). Clinical attachment loss (CAL) and probing pocket depth (PPD) were used to measure the severity of the periodontal disease. TOAST criteria were used to determine the ischemic stroke etiology, and the NIH Stroke Scale (NIHSS) was used to determine the ischemic stroke severity. A follow-up survey found that vascular incidents recurred.

Results

HPD individuals exhibited a higher median NIHSS (eight) than LPD patients (seven) in a subset of stroke population (N=23). Thirty-eight cardiovascular events occurred in the first three months after enrollment, including 23 strokes and seven TIAs, and five myocardial infarctions(MIs). There were three deaths from vascular causes. There was a non-significant association between PD and composite vascular events (HR 1.06, 95% CI, 1.03 to 1.09, p=0.71). Compound vascular events were not related to severe HPD (HR 1.31, 95 % CI 0.54 to 3.16, p=0.07).

Conclusion

In stroke/TIA patients, there is no link between high periodontal disease and recurrent vascular episodes. The proportions of stroke subtypes were not substantially different between HPD and LPD.

## Introduction

In terms of stroke/ transient ischemic attack (TIA) patients' recurrence risk, little is known about the relationship between periodontal disease and cerebrovascular disease (CVD). The burden of stroke is increasing in India; stroke is now the fourth leading cause of death and the fifth leading cause of disability [1]. It is possible that as one gets older, the various risk factors intersect, or there is a predisposition to atherosclerosis; the small and large arteries develop more complex lesions with bleeding or thrombosis [2]. When the tissues and bone that support teeth become involved with periodontal disease (PD), tooth loss occurs. The bacteremia caused by periodontal disease is closely correlated to its severity [3,4]. Routine activities like mastication may lead to periodontal bacteria entering the circulation [5,6]. The main arteries and veins of the body have been identified to harbor bacteria that cause periodontal disease [7,8]. Insulin resistance, low-density lipoprotein (LDL) oxidation, LDL aggregation, foam cell formation, and inhibition of endothelial nitric oxide synthase may be caused by inflammation caused by these bacteria [9]. Atherosclerosis and cardiovascular disease (including stroke) are both exacerbated by periodontal disease [10, 11]. Chronic oral infections like periodontitis may be an under-recognized modifiable risk factor that may be linked to stroke via several pathways. Periodontal disease and stroke were discovered to be linked in 1989. Since then, several studies have investigated the link between periodontal disease and stroke using various epidemiological methodologies, such as cross-sectional and case-control studies. According to earlier studies, those who have had an ischemic stroke or a transient ischemic attack are more likely to develop periodontal disease and suffer recurrent vascular episodes [12-14]. The present study was taken up with the aim of determining the association between recurrent vascular events and periodontal disease (PD) and to determine the extent and severity of periodontal disease among patients with ischemic stroke/TIA.

## Materials and methods

In this longitudinal, prospective cohort study, stroke/TIA patients over the age of 18 who presented to the department of medicine of Dr. Radhakrishnan Government Medical College over two years were evaluated for inclusion in the study. Out of 290 patients with stroke/TIA, 153 patients satisfied all the criteria and agreed to participate in the study. Before the beginning of the study, written informed consent was obtained from each patient. All procedures were carried out in accordance with the ethical rules and the principles of the Declaration of Helsinki. 

Acute ischemic stroke/TIA patients over 18 years of age were included in the study. Critically ill patients or patients with Glasgow Coma Scale <13, not being able to give informed consent or participate during a dental examination, or being identified with illnesses that necessitate preventive antibiotic treatment before the dental examination, subjects under the age of 18, life expectancy less than a year due to underlying medical condition, unreliable periodontal disease (PD) evaluation (low number of teeth), recent sickness, and/or antibiotic usage (within 30 days) were excluded from the study. Risk factors and etiologic stroke subtypes were examined in all individuals. To rule out intracerebral and subarachnoid hemorrhages, computed tomography/magnetic resonance imaging (CT/MRI) of the brain was utilized. Protocol-mandated tests for PD, and monitoring for recurrent vascular events was done in all subjects.

Data collection

Risk variables, laboratory tests, and subtypes of TIA/stroke were evaluated at the beginning of the study. Risk factors for vascular and periodontal diseases, as well as the use of medication and dental care (such as the frequency with which people brush their teeth and how often they visit the dentist, as well as any previous dental treatments), were all assessed using a questionnaire to determine the prevalence of different risk factors, such as hypertension and type 2 diabetes. Patients were examined by the investigator in the dental unit with a dental light, compressed air, and an optical mirror, as is standard practice in dentistry. The periodontal health of all teeth except the third molars were assessed using the Gingival Index (GI), probing pocket depth (PPD), and the number of missing teeth. Clinical attachment level (CAL, in millimeters) was used to assess periodontal health. Subjects were classified into low periodontal disease (LPD) and high periodontal disease (HPD). Subjects with mild periodontitis were considered LPD, and the subjects that had moderate or severe periodontitis were considered as HPD based on the presence and severity of CAL and PPD. The clinical case definition of periodontitis for severe periodontitis was considered ≥2 interproximal sites with CAL ≥4 (not on the same tooth) and ≥1 interproximal site with PPD ≥5; for moderate periodontitis was ≥2 interproximal sites with CAL ≥4 (not on the same tooth) or ≥2 interproximal site with PPD ≥5; for mild periodontitis was ≥2 interproximal sites with ≥3 mm CAL and ≥2 interproximal sites with ≥4 mm PPD (not on the same tooth) or one site with ≥5 mm [11]. Ischemic stroke etiology was classified using the Trial of Org 10172 in Acute Stroke Treatment (TOAST) criteria [15]. The initial ischemic stroke severity was measured using the National Institutes of Health Stroke Scale (NIHSS) [16] performed on the presentation of the patient to the hospital by an investigator. Monthly phone calls were made by the investigator for a median of 12 months after registration to monitor for vascular events such as a stroke or myocardial infarction (MI), as well as to check on the patient's well-being in the case of vascular death. On the follow-up phone calls, the participants were questioned about hospitalization, MI or recurrent stroke/TIA, emergency visits, and outpatient procedures that they underwent. A check of the medical records was done for patients who had an outcome event to ensure that the information was accurate. Each patient's death date and reason were documented for those who died during the trial.

Statistical analysis

The data was shown as either a percentage (%) or a mean (SD), depending on the situation. In order to compare the standard deviations of all continuous which have a normal distribution, a t-test was utilized. The median was used to compare non-uniformly distributed data in the Mann-Whitney U-test (interquartile range). When determining whether or not there were any differences between groups, the χ2 test was applied. A level of p≤0.05 was considered statistically significant, and p≤0.001 was noted as highly significant. Periodontal examination was performed on the mesial, distal, and midportions of teeth. Data was presented as number (%) or mean (SD) as appropriate. Mean values and interproximal locations were used to quantify probing depth (PD) and loss of attachment (AL), and PD and AL that were at or above the stated cut-off values were recorded (AL). The median and interquartile range were determined using the Mann-Whitney-U test (IQR). The Kaplan-Meier product limit technique and log-rank were used to determine the cumulative event-free rates for the time to composite vascular events (stroke, TIA, MI, and vascular death). After correcting for major confounders, risk factors for composite vascular events were estimated using the Cox proportional hazards analysis technique indicated below. All study participants had complete information on their exposure and result (recurrence of vascular events). In previous research, it was assumed that individuals with stroke or TIA had a high risk of composite vascular events [12,13], so the sample size was calculated based on this assumption. The estimated sample size was 200 subjects, and the log-rank test required a minimum of 80 participants in each exposure group to be possible to perceive a 15% difference in composite vascular events with 90% accuracy (a prior hypothesis). The type I (alpha) error rate was determined to be set at 0.05.

## Results

One hundred fifty-three patients completed a full periodontal examination (mean ± standard deviation 2±1 months). Seventy-two percent of the participants were males, 25.9% of the participants were alcohol abusers, and 73.1% of the participants never smoked. Table 1 shows the frequency distributions for the whole cohort, categorized by gender. Table 2 compares the baseline characteristics of patients with HPD (N=55) and individuals with LPD (N=98). Patients with HPD were significantly more likely to be older, with a P-value of 0.001. With a greater prevalence of hypertension and type 2 diabetes (p=0.07), men account for more than half of subjects with low periodontal disease (LPD). However, the difference between stroke and TIA patients was not statistically significant (62.5 percent vs. 20.8 percent, p=0.702). A p-value of 0.705 suggests that the percentage of stroke subtypes is not significantly different between HPD and LPD. LVD (Large Vessel Disease) was shown to be substantially more widespread in individuals with HPD (55 percent vs. 7.5 percent, p=0.001) than in those without HPD. In comparison to the LPD group, the HPD group's median NIHSS was higher (p=0.12), although the difference was not statistically significant.

**Table 1:** Gender distribution of variables

Variable	Male (%)	Female (%)	Total
Hypertension	141 (76.6)	43 (23.4)	184
Diabetes mellitus	65 (74.71)	22 ((25.29)	87
Dyslipidemia	50 (73.5)	18 (26.5)	68
Heart disease	11 (45.8)	13 (54.2)	24
Smokers	75 (96.1)	3 (3.9)	78
Alcohol abuse	75 (100)	0	75
Ischemic stroke	159 (70.9)	65 (29.1)	224
Hemorrhagic stroke	51 (77.2)	15 (22.3)	66

**Table 2:** Baseline characteristics of study participants *Normally distributed continuous values are summarized as mean ± standard deviation and compared using t-test; **p<0.05 is considered statistically significant; ***Continuous variables that are not normally distributed are depicted as median (inter-quartile range) and compared using Mann-Whitney U-test HPD - high periodontal disease, LPD - low periodontal disease, LVD - large vessel disease, SVD - small vessel disease, TOAST - Trial of Org 10172 in Acute Stroke Treatment, NIHSS - National Institutes of Health Stroke Scale

Characteristic			HPD (n=55)	LPD (n=98)	p-value
Age*			59.40±12.21	53.03±12.82	0.003**
Male			45 (40.9%)	65 (59.1%)	0.41
Hypertension			35 (35.7%)	63 (64.3%)	0.93
Diabetes			19 (47.5%)	21 ( 52.5%)	0.07
Dyslipidaemia			16 ( 44.4%)	20 (55.6%)	0.22
Smoking			17 (44.7%)	21 (55.3%)	0.19
Heart disease			3 (25%)	9 (75%)	0.41
Alcohol			17 (43.6%)	22 (56.45%)	0.24
Type of stroke	Ischemic		40 (35.1%)	74 (64.9%)	0.702
	Hemorrhagic		15 (38.1%)	24 (61.5%)	
TOAST classification		LVD	22 (55%)	15 (20.3%)	0.005**
		Cardioembolic	3 (7.5%)	9 (12.2%)	
		SVD	9 (22.5%)	27 (36.5%)	
		Other	1 (2.5%)	2 (2.7%)	
		Unknown	5 (12.5%)	21 (28.4%)	
NIHSS classification***			8 (6, 10)	7 (4, 10)	0.12

**Figure 1:**
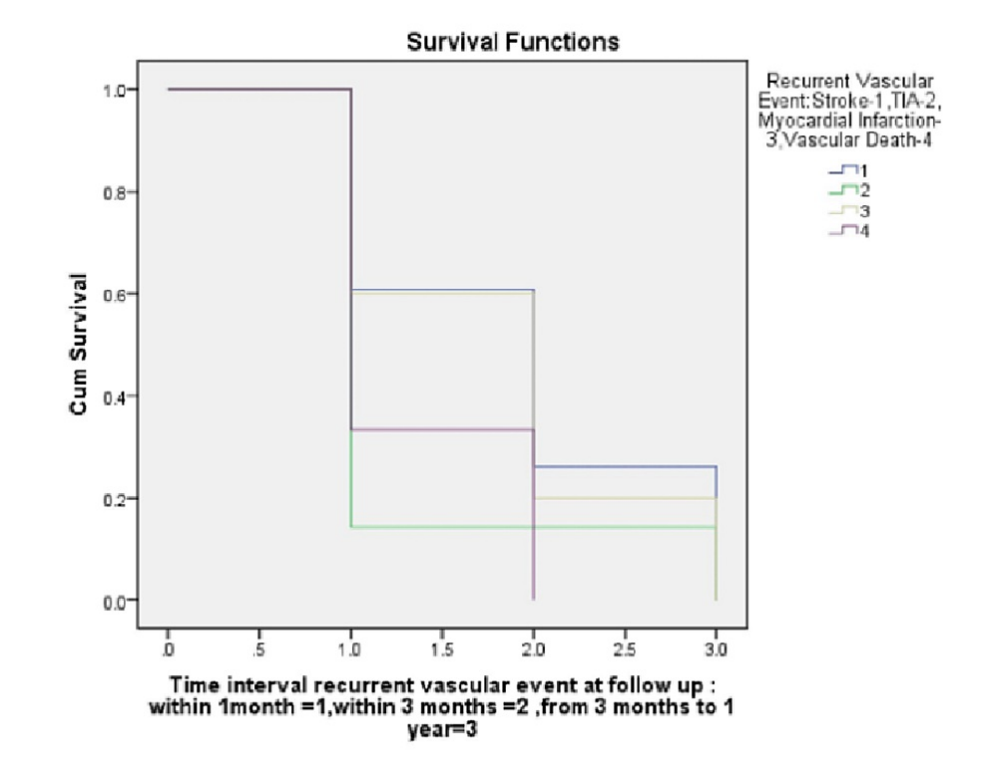
Kaplan-Meier survival curve TIA - transient ischemic attack

Over a median of three months from enrollment, 38 patients exhibited vascular events, including 30 with cerebrovascular events (23 strokes and seven TIAs), five MIs, and three vascular deaths. Out of a total of 55 patients with HPD, 24 (43.6%) patients with a severe form of periodontitis had vascular events, including twenty with cerebrovascular events (15 strokes and five TIAs), two MIs, and two vascular deaths. Among the 31 patients with a moderate form of periodontitis, 14 (45.1%) had vascular events, including ten stroke/TIA, three MI, and one vascular death over the same period of follow-up. No statistically significant differences were found between the two Kaplan-Meier survival curves (p=0.32) shown in Figure 1. PD and composite vascular outcomes correlations were not influenced by any other factors, except for age and stroke status. After correcting for these confounding variables, there was no longer a significant link between PD and composite vascular events (HR 1.06, 95 percent CI 1.03 to 1.09, p=0.71; Table 3). As previously stated, patients with severe HPD were older, more male, and more likely to develop LVD (Table 2). There was no link between periodontal disease and the composite of vascular events once these factors were taken into account (HR 1.31, 95 percent CI 0.54 to 3.16, p=0.05). There was no correlation between severe hypertension and composite vascular events in a Cox model with many covariates (HR 1.31, 95 percent CI 0.54-3.16, p=0.07), according to the model (Table 4).

**Table 3:** Distribution of recurrent vascular events *p<0.05 is considered statistically significant

Recurrent vascular event	Number of events		
Stroke	23		
Transient ischemic attack	7		
Myocardial infarction	5		
Vascular death	3		
Total	38		
Overall comparison			
	Chi-square	df	Sig.
Log Rank (Mantel-Cox)	3.480	3	0.323

**Table 4:** Hazard ratio for recurrent vascular events in stroke/transient ischemic attack (TIA) patients

	Hazard ratio
Adjusted for age and stroke status	1.06 (1.03-1.09)
Adjusted for age and stroke subtype	1.31 (0.54-3.16)
Multivariate adjustment	1.31 (0.54-3.16)

## Discussion

The findings of the present study indicate that in stroke/TIA patients, HPD is not associated with an increased risk of recurrent vascular events. The results indicate that stroke patients were more likely to have a severe form of periodontal disease (PD) than TIA patients (62.5 percent vs. 20.8 percent). Patients with HPD were more likely to develop large vessel disease (LVD) than those with LPD, although the percentages of stroke subtypes did not seem to change (55 percent vs. 7.5 percent). In contrast to Lafon et al.'s meta-analysis of cohort studies, which showed that periodontitis is associated with the occurrence of stroke, the results of this research study show that stroke incidence (death or non-death, ischemic or hemorrhagic), as well as baseline periodontal status, are not related. Similar results have been found in a recent study in which periodontal disease and stroke have no connection (ischemic and hemorrhagic strokes combined) [17]. Even though the link between periodontal disease, cardiovascular illness, and ischemic stroke has been investigated recently, the relationship between the two is well-established [18]. According to the results of various studies, stroke is linked to periodontitis and tooth loss. An increase in the risk of stroke was seen in the subjects with periodontitis in the review by Ross et al. (Relative risk of 1.63 (1.25, 2.00)) [19]. PD is related to ischemic stroke in a significant way in the recent research [20]. In a study seeking a correlation between PD and ischemic stroke, dental pathogenic bacteria were isolated from carotid plaque and correlated with carotid intimal medial thickness [21,22]. Gram-negative bacteria are associated with an increase in systemic inflammatory markers, which are linked to atherosclerosis and stroke [12]. Detection and management of risk factors may lessen the impact of a stroke.

Periodontal disease has been associated with vascular diseases in several studies [22-27]. After considering significant confounders, including gender, smoking, habit, and socioeconomic status, the majority of these clinical and epidemiologic research indicated a 20%-30% association between periodontal disease and cardiovascular outcomes [27]. Out of all the variables in the present study, only age and stroke status substantially did not confound the association between PD and composite vascular outcome association. When these confounders were taken into account, the association between periodontal disease and composite vascular events remained insignificant. Figure 1 shows a lack of significant distinction in survival curves between HPD and LPD patients three months after the periodontal evaluation in the Kaplan-Meier plots. Andriankaja et al. [28] and Hyman et al. [29] showed evidence of a link between PD and MI in both sexes regardless of the putative confounding impact of smoking in both genders in this study. Research by Sen et al. [30] examined whether PD was connected with aortic arch atheroma (AA). Patients with a history of stroke or TIA had an increased chance of recurrence if they had a severe form of periodontal disease. There is a need for more studies to validate the association and to investigate if the treatment of PD can lessen the pace of plaque advancement and the number of recurrent cardiovascular events. There are certain limitations to this research. As a result of this study's small sample size, it is not possible to test whether TIA or particular stroke subtypes are associated with each other. Information is lacking in the study about the role of inflammatory markers, which can be hypothesized as a plausible mechanism linking periodontal disease to cardiovascular disease (CVD).

## Conclusions

Stroke/TIA patients have more probability of suffering from an advanced form of periodontal disease. After correcting for potential confounding variables, no association could be identified between periodontal disease and a composite of vascular events. For stroke/TIA victims, periodontal disease did not affect the recurrence rate of vascular events. The proportions of different types of stroke were virtually identical.
